# Distribution of group B streptococci serotypes on women nasopharynx

**DOI:** 10.1016/j.bjid.2024.104469

**Published:** 2024-11-21

**Authors:** Moises Dantas Cartaxo de Abreu Pereira, Fabrine Felipe Hilário, Eloiza Helena Campana, Eduardo Sergio Soares Sousa, Vinicius Pietta Perez

**Affiliations:** a Universidade Federal da Paraíba (UFPB), Centro de Ciências Médicas (CCM), Mestrado Profissional em Saúde da Família, João Pessoa, PB, Brazil; b Universidade Federal da Paraíba (UFPB), Centro de Ciências Médicas (CCM), Laboratório de Biologia Molecular (LaBiMoL), João Pessoa, PB, Brazil; c Universidade Federal da Paraíba (UFPB), Centro de Ciências da Saúde (CCS), Núcleo de Medicina Tropical (NUMETROP), João Pessoa, PB, Brazil

**Keywords:** *Streptococcus agalactiae*, Neonatal infection, Capsular polysaccharide

## Abstract

Group B Streptococcus (GBS) is a significant pathogen responsible for neonatal infections, primarily transmitted through maternal carriage. However, current preventive strategies, such as intrapartum antibiotic prophylaxis, present limitations and are ineffective in preventing late-onset neonatal infections. This study aimed to assess the prevalence and serotype distribution of GBS in the nasopharynx of women of reproductive age, providing data to the potential implementation of a novel hexavalent capsular vaccine (GBS6). Nasopharyngeal swabs were collected from 500 women and analyzed using a qPCR assay targeting the *cfb* gene to detect GBS and the *cps* locus. GBS was identified in 7.4 % of patients, with serotype Ia being the most prevalent. Other serotypes detected included II, V, Ib, III, and IV. These findings suggest that the nasopharynx may act as a reservoir for GBS in women of reproductive age. The results also highlight the importance of developing preventive strategies focused on upper respiratory tract colonization. Additionally, the potential introduction of the GBS6 vaccine could provide significant coverage against circulating GBS serotypes.

*Streptococcus agalactiae,* or Group B Streptococcus (GBS), is a member of the human microbiota that primarily colonizes the gastrointestinal and genitourinary tracts and, less frequently, the oropharynx.^1^ GBS is a significant opportunistic pathogen associated with several infections in neonates, young infants, mothers, and immunocompromised patients.^2^

The neonatal infections can manifest as Early-Onset Infections (EOI), occurring in the first week of life, primarily acquired during delivery through colonization of the maternal genitourinary tract. To prevent EOI, the administration of Intrapartum Antibiotic Prophylaxis (IAP) to colonized mothers during labor is recommended.^3^ Late-Onset Infections (LOI) occur between the first week and three months of age and can result from GBS transmission from the maternal microbiota or environmental sources.^1^ Currently, there is no effective measure to prevent LOI. Despite the reduction of EOI with IAP programs, challenges related to GBS infection persist, and the development of a novel hexavalent vaccine against GBS (GBS6) capsular saccharides (serotypes Ia, Ib, II, III, IV and V) remains a promising alternative for preventing LOI.^3^^,^^4^

Since maternal colonization is the principal route of GBS transmission, the colonization of the upper respiratory tract could be a reservoir of GBS, potentially playing a role in maternal transmission to neonates after delivery. There is limited available data regarding the epidemiology of GBS in the nasopharynx and this study investigated the prevalence of GBS in women of reproductive age and evaluated the serotype distribution, providing important information for the future implementation of GBS capsular vaccines.

Nasopharyngeal swabs collected from women of reproductive age (14‒45 years) in Phosphate-Buffered Saline (PBS) for detection of viral pathogens at a hospital in João Pessoa City, Brazil, between July to September 2020 and stored at −70 °C were included in this study. Informed consent was obtained from patients and all procedures were performed in compliance with laws and institutional guidelines. The study protocol was approved by the Institutional Ethics Committee (5.488.788, on June 24, 2022). For each sample, 0.2 mL of PBS was incubated in a solution of lysozyme (5 mg/mL) and mutanolysin (25 U/mL) (Sigma-Aldrich, USA) for 1 hour at 37 °C for bacterial lysis. The DNA extraction/purification was carried out by the automated Maxwell RSC System using the Maxwell TNA kit (Promega, USA), according to manufacturer instruction, and stored at −70 °C before qPCR amplifications.

The presence of GBS on nasopharyngeal samples was determined by amplification of *cfb* gene in a qPCR assay^5^ Briefly, reactions were performed using 5 µL of the extracted/purified DNA in a final volume of 20 µL using GoTaq Probe qPCR System (Promega, USA) and a final concentration of 200 nM of primers and probe. Amplifications were carried in a QuantStudio3 Real Time PCR System (Thermo-Fisher Scientific, USA) using the following cycling parameters: 95 °C for 2 min, followed by 40 cycles of 95 °C for 15 s and 60 °C for 60 s. All reactions were performed in duplicates and during each analysis, a negative and positive control were used.

The determination of capsular type (Ia, Ib, II, III, IV, V, VI, VII, VIII and IX) in samples positive for the presence of GBS was determined by amplification of *cps* locus using ten distinct qPCR reaction, in all samples, as previously described.^6^ The reactions were performed in duplicate using 5 µL of the extracted/purified DNA in a final volume of 20 µL using GoTaq Probe qPCR System (Promega, USA) and a final concentration of 100 nM of primers and probe. The cycling and analyze parameters were the same for the amplification of *cfb* gene.

The statistical analysis of the data includes absolute and relative frequencies to describe the distribution of variables. The fisher-exact test for the comparison between groups was carried out using SPSS 20.0 for Mac OS (IBM corporation) with a significance of *p* < 0.05.

A total of 500, non-duplicate, nasopharyngeal swabs from women of reproductive age were evaluated for the presence of GBS. Amplification of the GBS *cfb* gene was observed in 37 patients, indicating a prevalence of 7.4 % for upper respiratory tract colonization in our population. Table 1 shows the prevalence according to women's age, and there is no statistical difference between age groups colonization in our sample (*p* = 0.724).

**Table 1:** Prevalence of *Streptococcus agalactiae* (GBS) colonization and serotypes in the nasopharynx of women of reproductive age.

**Age (years)**	**GBS (%)**		**Serotype**						
	**Negative**	**Positive**	**Ia**	**Ib**	**II**	**III**	**IV**	**V**	**NT**
< 25	59 (92.2)	5 (7.8)	2	0	2	0	0	1	0
25‒34	154 (91.7)	14 (8.3)	3	0	1	1	1	2	6
35‒39	123 (91.8)	11 (8.2)	3	0	1	0	0	1	6
≥ 40	127 (94.8)	7 (5.2)	2	1	2	0	0	0	2
Total	463 (92.6)	37 (7.4)	10	1	6	1	1	4	14

GBS, Group B Streptococci, *Streptococcus agalactiae*; NT, Non-Typeable.

Among the samples positive for the *cfb* gene, determination of the *cps* locus was possible in 23 (62.16 %) samples. Fourteen samples did not amplify any of the ten *cps* loci tested and were therefore classified as non-typeable. Six distinct serotypes were detected in our population, with no sample testing positive for more than one serotype. Serotype Ia was the most prevalent (43.49 %, *n* = 10), followed by serotypes II (26.09 %, *n* = 6), V (17.39 %, *n* = 4), Ib (4.35 %, *n* = 1), III (4.35 %, *n* = 1), and IV (4.35 %, *n* = 1), Fig. 1.

**Fig. 1:**
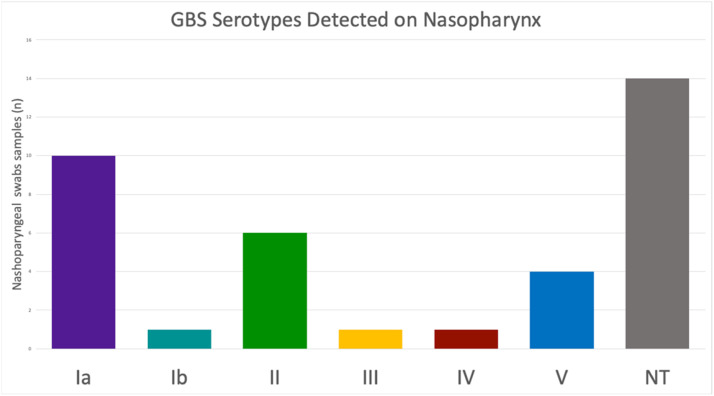
Distribution of *Streptococcus agalactiae* (GBS) serotypes detected direct from nasopharyngeal swabs from women of reproductive age in João Pessoa city, Brazil. NT, Non-Typeable.

The implementation of IAP has significantly decreased the incidence of EOD in regions with established control programs. However, the burden of LOD remains stable or has even increased following the implementation of IAP strategies.^4^ Furthermore, the transmission routes of GBS related to LOD are not clearly understood, with breast milk and close facial contact potentially involved.^7^ Indeed, there is limited evidence regarding the colonization of the upper respiratory tract in mothers and adults. A meta-analysis estimated a prevalence of 9.2 % among North American and European adults.^8^ In Africa, a maternal colonization rate of 1.1 % was observed over a period of 28 days after delivery, with no reported relationship between maternal nasopharyngeal carriage and newborn colonization.^9^ A study evaluating oropharyngeal colonization of GBS among neonatal close contacts showed an overall prevalence of 23.1 %.^7^ The high variation observed among these studies could be related to methodological differences such as the sampling site (oropharynx, nasopharynx, nose, oral), processing methods (culture or PCR), and prior use of antibiotics. In our population, a prevalence of 7.4 % was observed using a qPCR assay. Since qPCR detects specific bacterial genomic fragments, it is more sensitive and can detect low bacterial yields and is less affected by prior antibiotic use. However, its results may not directly indicate the presence of viable cells.

The rates of maternal vaginal/anal colonization vary globally, with an estimated global prevalence of 18 %, ranging from 11 % to 35 %, and a prevalence of 15.7 % in South America.^10^ According to the American College of Obstetricians and Gynecologists, young maternal age is a risk factor associated with EOD.^11^ However, the risk of vaginal/anal colonization has been shown to increase with maternal age^12^, ^13^, ^14^ and the elevated risk among young women may be linked to reduced access to healthcare services.^13^ Nonetheless, in our population, no significant differences were observed in the distribution of nasopharyngeal GBS colonization across age groups.

The capsule plays a central role in GBS virulence, with ten antigenically and structurally distinct polysaccharide compositions described (Ia, Ib, II to IX). Serotype Ia is most frequently recovered from pregnant women, while serotype III is highly associated with invasive diseases in newborns.^1^^,^^2^ Furthermore, the regional prevalence of serotypes is not uniform. A systematic review covering the period from 2001 to 2018 showed increasing trends of serotype IV in developed regions. In other regions, serotypes III, V, and VI-IX have shown an increase in maternal colonization, with serotype III being predominant in LOD throughout the period.^15^ In recent years, an increasing trend of serotypes V and Ib and a decreasing trend of serotypes II and III have been reported among pregnant women in Brazil.^16^ An evaluation of isolates obtained from the adult oropharynx in the USA showed that the most frequent serotype was III, followed by V, Ib, and II.^7^ In our population, six serotypes were found (Ia, Ib, II, III, and V) in nasopharyngeal samples, and the GBS6 vaccine in development might provide high coverage for GBS obtained from the upper respiratory tract of women.^3^

However, fourteen samples in our population were non-typeable. The GBS *cps* locus is a chromosomal region that harbors the genes involved in capsule synthesis, similar to the pneumococcal capsule. The locus contains several conserved genes and distinct transferase organizations for each serotype.^17^ Molecular capsular typing methods allow the determination of GBS serotypes directly from clinical specimens without the need for prior bacterial isolation. Although, in clinical samples, the bacterial genome could be fragmented, reducing the availability of suitable target sequences for amplification, resulting in reduced assay sensitivity.

In conclusion, the presence of GBS in the maternal upper respiratory tract may be an important route of transmission to neonates, consequently contributing to neonatal infections. We observed that the nasopharynx may serve as a significant reservoir for GBS in women of reproductive age. Furthermore, the GBS6 hexavalent capsular polysaccharide vaccine could provide high coverage against GBS in our population.

## Conflicts of interest

The authors declare no conflicts of interest.
